# All-Atom Molecular Dynamics of Pure Water–Methane Gas Hydrate Systems under Pre-Nucleation Conditions: A Direct Comparison between Experiments and Simulations of Transport Properties for the Tip4p/Ice Water Model

**DOI:** 10.3390/molecules27155019

**Published:** 2022-08-07

**Authors:** André Guerra, Samuel Mathews, Milan Marić, Phillip Servio, Alejandro D. Rey

**Affiliations:** Department of Chemical Engineering, McGill University, Montréal, QC H3A 0G, Canada

**Keywords:** water, methane, hydrates, viscosity, diffusivity, molecular dynamics

## Abstract

(1) Background: New technologies involving gas hydrates under pre-nucleation conditions such as gas separations and storage have become more prominent. This has necessitated the characterization and modeling of the transport properties of such systems. (2) Methodology: This work explored methane hydrate systems under pre-nucleation conditions. All-atom molecular dynamics simulations were used to quantify the performance of the TIP4P/2005 and TIP4P/Ice water models to predict the viscosity, diffusivity, and thermal conductivity using various formulations. (3) Results: Molecular simulation equilibrium was robustly demonstrated using various measures. The Green–Kubo estimation of viscosity outperformed other formulations when combined with TIP4P/Ice, and the same combination outperformed all TIP4P/2005 formulations. The Green–Kubo TIP4P/Ice estimation of viscosity overestimates (by 84% on average) the viscosity of methane hydrate systems under pre-nucleation conditions across all pressures considered (0–5 MPag). The presence of methane was found to increase the average number of hydrogen bonds over time (6.7–7.8%). TIP4P/Ice methane systems were also found to have 16–19% longer hydrogen bond lifetimes over pure water systems. (4) Conclusion: An inherent limitation in the current water force field for its application in the context of transport properties estimations for methane gas hydrate systems. A re-parametrization of the current force field is suggested as a starting point. Until then, this work may serve as a characterization of the deviance in viscosity prediction.

## 1. Introduction

Gas hydrate structures form under low temperatures and elevated pressures. Under these conditions, the liquid-to-solid phase transition of water favors the formation of a gas hydrate solid structure over that of hexagonal (Ih) ice. During the phase transition, a “guest” gas species dissolved in water can become trapped in cages that form in the molecular lattice. Gas hydrates have three common types of structure, namely sI, sII, and sH, which differ in their geometric morphology and the sizes of the cages that they form [1]. Methane gas mainly forms sI hydrates as its molecular radius allows it to fit in the cages of this structure. The guest molecule’s presence in the cage stabilizes the structure by lowering its potential energy [1,2].

Operational conditions in the oil and gas industry and the prevalence of water and gas in multi-phase systems such as transportation pipelines and drill wells are conducive to gas hydrate formation [3]. As a result, historically, gas hydrate research has focused on solving physical, economic, and engineering issues common in the oil and gas industry [4]. The flow assurance of oil and gas systems has been the primary focus, and thus has motivated research into inhibitor additives to prevent or slow the formation of gas hydrates in flow lines [5,6,7,8,9,10,11]. Moreover, the rheological properties of these systems have been of interest [12,13,14,15,16,17,18]. These studies have mostly involved oil-in-water systems to reflect applications in the oil and gas industry.

Gas hydrates have a gas storage capacity of up to 180:1 gas-to-liquid volume ratio [1,19]. Additionally, the mixtures of gases with different molecular radii can be separated based on the size selectivity of the cages that form in gas hydrate structures. New technologies that take advantage of the formation and physical properties of gas hydrates have been proposed. These include gas separations [19,20], pre- and post-combustion carbon capture [21,22,23], the transport and storage of natural gas [24,25], and the desalination of water [26]. These emerging applications have led to research on promoter additives such as graphene nanoflake and multi-walled carbon nanotube nanofluids [27,28,29]. Many of the technologies above involve the continuous flow of pre-nucleation and nucleating gas hydrate systems that require to be maintained in the flow state for operation and involve aqueous systems (no oil). This has led to interest in characterizing the rheological properties of such systems. Rheological phase diagrams of methane and carbon dioxide hydrates systems in pure water have been recently developed [30]. However, the experimental limitations (kinetic, mass diffusion, and heat effects) of working with gas hydrates in shear rheometers that reduce the feasibility of such rheological work have also been identified [30]. Moreover, the addition of plasma-functionalized carbon nanotube nanofluid promoters to methane hydrate systems has been reported, suggesting molecular scale phenomena as a cause of non-Einsteinian dynamic viscosity effects [31].

Computational methods have enabled the investigation of multiple material properties of gas hydrates. Density functional theory (DFT) has been used to characterize the piezoelasticity and stability limits of hydrate structures [32,33] and investigate the vibration spectra of structure II and H as a method to predict the Young’s modulus of these hydrate structures [34,35]. DFT and phonon calculations have been used for the prediction of the thermal properties of methane, ethane, ethylene oxide, carbon dioxide, and empty sI and hexagonal ice (Ih) structures [36]. Molecular dynamics (MD) has also been a promising tool to investigate gas hydrate phenomena. It has been used to explore gas hydrate interfacial thickness and tension with high agreement to experimental results [37]. Additionally, MD has been used to investigate methane hydrate growth with impingement, which led to the observation of some cages being occupied by two methane molecules at the same time [38]. Moreover, MD has been previously used to predict the transport properties of water [39,40], methane, and carbon dioxide systems [41]. A comprehensive list of best practices for the calculation of transport properties by molecular dynamics has been compiled by Maginn et al. [42]. The computational estimation of the transport properties of pre-nucleation methane hydrate systems via MD is a promising alternative to overcome the limitations in the experimental rheology work of gas hydrates [30,31].

Molecular dynamics simulations offer a computational methodology for the solution of Newton’s equations of motions to represent atomic (and molecular) trajectories and interactions given force field potentials. Force fields are parametrized models designed to represent potential energy in an atomic system, which is a function of the individual atomic positions [43]. The total potential energy of the system represented by the force field includes bonded and non-bonded interactions. Potential energy from bonded interactions arise from changes in bond length, bond angle, and bond torsion along dihedrals. These tend to be modeled as harmonic oscillators. The potential energy from non-bonded interactions arise from electrostatic and repulsive (van der Waals) forces between non-bonded atoms. Coulombic pair-wise interactions commonly represent the electrostatic interactions in the system, while Lennard-Jones 12/6 potentials are used to model repulsive forces. The recursive solution of molecular systems and the application of statistical mechanical principles allows MD simulators to predict macroscopic properties such as viscosity, diffusivity, and thermal conductivity.

Molecular dynamics has been used a tool to study gas hydrates phenomena in multiple scenarios. Simulations have considered the formation/nucleation [44,45,46,47,48,49,50,51,52] and dissociation [53,54,55,56,57,58,59] of gas hydrates, guest gas mobility between cages and displacement by different species [60,61,62,63], surface effects on nucleation [64,65,66,67,68], the inhibition mechanisms of polymers such as poly(vinyl pyrrolidone) and poly(vinyl caprolactam) [69,70,71,72], as Qi et al. conducted a recent review of studies utilizing molecular simulations investigate gas hydrate phenomena [73]. However, the examination of the transport properties of methane hydrates systems under pre-nucleation conditions remains mainly unexplored, especially in pure water–methane systems. The work presented herein intends to examine the performance of the TIP4P/2005 and TIP4P/Ice water models in the prediction of transport properties of pure water under near freezing conditions and of pre-nucleation methane gas hydrate systems. This work will use previously obtained experimental viscosity data [30] to compare and validate computational estimations. Multiple formulations for the calculation of viscosity are evaluated here and the system’s adherence to the Stokes–Einstein constant across temperatures is examined. A large molecular system (7641 atoms), a rigorous equilibration methodology, and bootstrapping statistics (with replacement) of calculated values is performed to ensure the confidence and physical meaningfulness of the results presented herein.

## 2. Material and Methods

### 2.1. Software Packages

Molecular dynamics (MD) simulations involve the modeling of system energy and atomic interactions by dynamically tracking atomic trajectories through Newton’s equations of motion. The MD simulations conducted in this work were performed using the Large-scale Atomic/Molecular Massively Parallel Simulator (LAMMPS) package [74]. This is a mature and widely used MD simulation package developed and maintained by Sandia National Laboratory and Temple University. The molecular systems simulated in this work were defined using PACKMOL [75] and Moltemplate [76] packages. PACKMOL is a packing optimization algorithm that uses a random position generator to place atoms and/or molecules in a simulation box within a specified geometry and tolerance of separation from each other (2.5 angstroms in this work). As a result of PACKMOL’s optimized molecular positioning algorithm, the system’s inter-molecular repulsive forces are reduced and thus so is its initial potential energy, making the initial MD simulation steps less computationally intensive. This is an improvement on the regular lattice structure that is normally produced from standard simulation box populating commands available in LAMMPS or Moltemplate. Moltemplate is a Python software package that facilitates the assignment of force field parameters to atoms and/or molecules in an MD system [76]. It was used in this work to assign atom masses, charges, and molecule bond and angle parameters associated with the Optimized Potentials for Liquid Simulations All-Atom (OPLS-AA) force field for water and methane molecules. Finally, the MDAnalysis is a molecular trajectory handling and analysis software. The hydrogen bond (H-bond) analyses and the all-to-all RMSD plots discussed below were performed with the aid of MDAnalysis.

### 2.2. Simulation Design

#### 2.2.1. Force Field

In this work, the Optimized Potentials for Liquid Simulations (OPLS) force field was used in its all-atom form (OPLS-AA) [77,78,79]. The all-atom form of the force field allows the explicit representation of hydrogen atoms in the system, as opposed to the coarse-grained version united-atom (UA) form of the force field, which lumps hydrogen atom parameters with their bonded carbon atom. The explicit representation of hydrogen atoms in the all-atom parameters allows for the improved modeling of the hydrogen interactions in liquid water systems which involve a high presence of hydrogen bonding, such as those simulated in this work. The OPLS-AA force field was used to model methane molecules simulated in this work. Additionally, two variations of the original four-site water model TIP4P [77] were used in this work-TIP4P/2005 [80] and TIP4P/Ice [81]. The four-site models allow for an all-atom representation and interaction between the water molecules and the OPLS-AA methane molecules.

#### 2.2.2. LAMMPS Input

The water systems simulated here used the TIP4P/2005 and TIP4P/Ice OPLS-AA four-site water models. The TIP4P/2005 model was developed to represent water under any condition in its entire phase diagram, while TIP4P/Ice is a re-parameterized version of the TIP4P/2005 model designed for an improved representation of water properties in its solid state [80,81]. The water systems simulated in this work consisted of 2547 molecules, which is one order of magnitude larger than the classic 256 molecule system normally simulated, which suffers from finite size effects [40]. The systems were represented in LAMMPS with *full* atom style and *lj/cut/tip4p/long* pairstyle with 8.5 *Å* OM site and Coulombic cutoff lengths. A *pppm/tip4p* kspace space style with a dimensionless relative force accuracy of 1 × 10${}^{-5}$ and Lorenz–Berthelot arithmetic mixing rules were applied. Bond and angle styles were set to *harmonic*, with water molecules bonds kept rigid via the *shake* command. Although PACKMOL was used to minimize inter-molecular repulsive forces during the initial system definition, an energy minimization procedure was run in LAMMPS prior to the start of the equilibration run to ensure that minimum energy was achieved. All tests required less than 500 time steps to achieve a local minimum of potential energy for the system. The short simulation time required to achieve this local minimum can be attributed to the usage of PACKMOL as a preliminary step to reduce repulsive forces through its packing optimization algorithm. The Verlet algorithm with a 2-femtosecond timestep was used to conduct the numerical integration of Newton’s equations of motion. Table 1 contains the detailed list of parameters for the original TIP4P force field developed by Jorgensen et al. [77], and the modified TIP4P/2005 and TIP4P/Ice used in this work. Finally, Table 2 presents the OPLS-AA parameters used to model methane molecules in this work [79].

#### 2.2.3. Methane Systems

The pre-nucleation gas hydrate systems simulated here are made up of two species—water and methane. According to the Gibbs phase rule, the two-phase system with two component species has exactly two degrees of freedom. These are taken up by the temperature and pressure conditions imposed on the simulation. As a result, the methane concentration of the liquid mixture is fixed at every condition. Concentrations can be determined by the calculation of the methane fugacity as modeled by the Trebble–Bishnoi [82] equation of state (EOS) in combination with the modified Henry’s law for higher pressures as presented by Krichevsky and Kasarnovsky [83] and the methane dissolution in water measurements by Lekvam and Bishnoi [84]. The Trebble–Bishnoi EOS is a widely used model and has been extensively described and explored so it will not be included here. The modified Henry’s law for higher pressures by Krichevsky and Kasarnovsky is presented in Equation (1).

(1) $$\ln\frac{f_{2}}{x_{2}}=\ln H_{2,1}+\frac{{\bar{\nu }}_{2}^{\infty }\left(P-P_{1}^{S}\right)}{RT}$$

In this relation, the gas species fugacity in the liquid is $f_{2}$, $H_{2,1}$ is Henry’s law constant, $x_{2}$ is the gas concentration in the liquid phase, $P_{1}^{S}$ is the partial saturation pressure of the liquid, ${\bar{\nu }}_{2}^{\infty }$ is the partial molar volume of the gas at infinite dilution, *R* is the gas constant, *P* is pressure, and *T* is the temperature. The fugacity of methane in water is calculated using the Trebble–Bishnoi EOS [82]. Lekvam and Bishnoi provide values for Henry’s constant, $H_{2,1}$, and the partial molar volume, ${\bar{\nu }}_{2}^{\infty }$, of methane for temperatures between 1.2 and 10.2 °C [84]. These values were linearly extrapolated to include the 0 ℃ conditions. The methane systems consisted of 1470–2547 molecules depending on the concentration calculated.

### 2.3. Equilibration Procedure

The equilibration methodology conducted in this work was based on recommendations by Maginn et al. [42] regarding the best practices for the MD estimations of transport properties [42]. The initial system temperatures were assigned to the molecules in the form of initial velocities based on the Maxwell–Boltzmann distribution. All molecular systems in this work were equilibrated using a series of simulations. First, the system was simulated using the isobaric-isothermal (NPT) ensemble for 25 ns. During this phase, the system’s potential energy and density were monitored to be in the expected range based on temperature and pressure conditions, and to evolve to be stabilized over the simulation time [80,81,85]. Once satisfactory stabilization was achieved, the system was simulated for 100 ns using the canonical (NVT) ensemble with a Nosé–Hoover thermostat. The release of the NPT ensemble’s barostat reduces the mechanical disturbances (volume corrections) imposed on the system by the ensemble dynamics to maintain constant pressure. This is an important step as the autocorrelation functions used to calculate transport properties, particularly the Green–Kubo velocity and pressure autocorrelations, are affected by these disturbances [42]. Additionally, the pressure and temperature damping factors used in this work were 1000*dt and 100*dt, respectively, where “dt” is the simulation timestep (2 femtoseconds). This was determined to be enough to allow the system to relax between iterations of the barostat and thermostat, and thus help reduce the disturbances introduced by the dynamics of the ensemble. During the NVT equilibration step, the pressure and density of the system is monitored to ensure that they did not dramatically deviate from their values at the end of the NPT equilibration step.

### 2.4. Replicate Production Runs

Ideally, the NVE ensemble would be used for production runs to minimize the disturbances introduced into the system by the dynamic thermostat and barostat algorithms in NVT and NPT ensembles. However, the temperature control can be insufficient in NVE ensembles, and can be inappropriate when simulating temperature-specific property data points. Additionally, in the context of dynamic viscosity and diffusivity estimations, it has been shown that predictions from NVT ensembles are indistinguishable from those obtained from NVE ensembles [86,87]. Thus, the production runs simulated in this work were performed using the NVT ensemble.

Once equilibration was satisfactorily achieved, ten replicate production runs per temperature–pressure condition considered were simulated, each with a different random number generator seed for the temperature (velocity) assignment according to the Maxwell distribution. These replicates were used to create a sample of ten production runs (*n* = 10) for statistical analysis. Bootstrap analyses (with replacement and *n* = 10,000) of samples with ten simulations per condition (*n* = 10) were performed on the replicate production runs. Bootstrap statistics can improve the reliability and accuracy of the transport property predictions, as the uncertainty in prediction is inversely proportional to the square root of the number of replicates ($u\propto 1/\sqrt{n}$) [88]. Furthermore, replicate runs benefit from the computational speed of parallel simulations versus one long simulation, which have been shown to converge to the same value [89,90]. Additionally, the random states generated by the temperature assignment described above have the effect of reducing the disturbances, or noise, on the Green–Kubo integral and the Einstein slope via the averaging of the disturbances that occur across the random states generated. This improves the reliability on the GK and Einstein transport property calculations, which will be described in the next section.

### 2.5. Transport Property Calculations

Molecular dynamics simulations produce mechanical property data of the systems simulated as they arise from the fundamental principles of statistical mechanics [74]. The fluctuations of mechanical properties such as pressure, velocity, or heat flux can be used to estimate transport properties of molecular systems. The equilibrium time autocorrelations of these mechanical variables can be used to calculate the viscosity, diffusivity, and thermal conductivity as described by the Green–Kubo (GK) integral definition [91,92]. Viscosity utilizes the elements of the pressure tensor (Equation (2)), while diffusivity uses the molecular velocities (Equation (3)). Additionally, thermal conductivity can also be estimated by the Green–Kubo formulation using the autocorrelation of the heat flux through surfaces normal to each dimension of the simulated system (Equation (4)).

(2) $$\eta_{GK}=\frac{V}{k_{B}T}\int_{0}^{\infty }\langle P_{\alpha \beta }\left(t\right)\cdot P_{\alpha \beta }\left(0\right)\rangle dt$$

(3) $$D_{GK}=\frac{1}{d_{\alpha }}\int_{0}^{\infty }dt\langle \frac{1}{N}\sum_{i=1}^{N}v\left(t\right)\cdot v\left(0\right)\rangle$$

(4) $$\kappa_{GK}=\frac{1}{d_{\alpha }}\frac{V}{k_{B}T^{2}}\int_{0}^{\infty }\langle J(t)\cdot J(0)\rangle dt$$

where $k_{B}$ is the Boltzmann constant, $d_{\alpha }$ is the number of dimensions in the simulation ($d_{\alpha }=3$ in this work), $P_{\alpha \beta }$ is an off-diagonal element of the pressure tensor, *v* is velocity, and *J* is the heat flux through a surface.

The Green–Kubo formulation for transport properties can be integrated and re-stated as a differential form, normally referred to as the Einstein (Eins) formulation. For instance, the Green–Kubo diffusivity equation can be re-expressed with the Einstein formulation as presented in Equation (5). The Einstein formulations tend to be preferred over the Green–Kubo ones due to the instability of the time autocorrelation of the respective mechanical property and the longer time requirement for convergence when compared to the slope used by the Einstein formulations [42]. This work explores the application of an additional formulation of viscosity, namely the Stokes–Einstein formulation (Equation (7)). This formulation utilizes the Stokes–Einstein relation for spherical particles in low Re flow (Equation (6)) and the Einstein formulation of diffusivity described above (Equation (5)).

(5) $$D_{Eins}=\frac{1}{6}\lim_{t\to \infty }\frac{d}{dt}\left(\right|r_{i}\left(t\right)-r_{i}\left(0\right){|}^{2})$$

(6) $$D=\frac{k_{B}T}{6\pi \eta r}$$

(7) $$\eta_{SE}=\frac{k_{B}Tdt}{\pi rMSD}$$

where $MSD\equiv |r_{i}\left(t\right)-r_{i}{\left(0\right)|}^{2}$ is the mean squared displacement of molecules, *r* is the molecular radius, and $r_{i}$ is the position of the *i*th molecule.

## 3. Results and Discussion

### 3.1. Molecular Simulation Design Considerations

The equilibration suggestions for the development of molecular simulations with the interest of performing transport property predictions presented by Maginn et al. [42] were followed in this work. The rigorous equilibration procedure ensured that simulations are physically meaningful and results are reliable. This work used multiple design considerations and simulation indicators to evaluate whether the simulated systems achieved equilibrium. The first consideration was the consecutive equilibration steps, which were discussed above as a series of simulations starting from an isobaric-isothermal (NPT) ensemble followed by a canonical (NVT) ensemble. During the consecutive simulations, significant simulation properties were used to determine the approach to equilibrium. Additionally, long-term indicators of equilibrium were used to confirm whether the simulated system achieved a diffuse regime. Finally, a discussion on the importance of H-bonds to the molecular simulation predictions of transport properties in water-rich systems is presented.

#### 3.1.1. Consecutive Equilibration Steps

**Figure 1:**
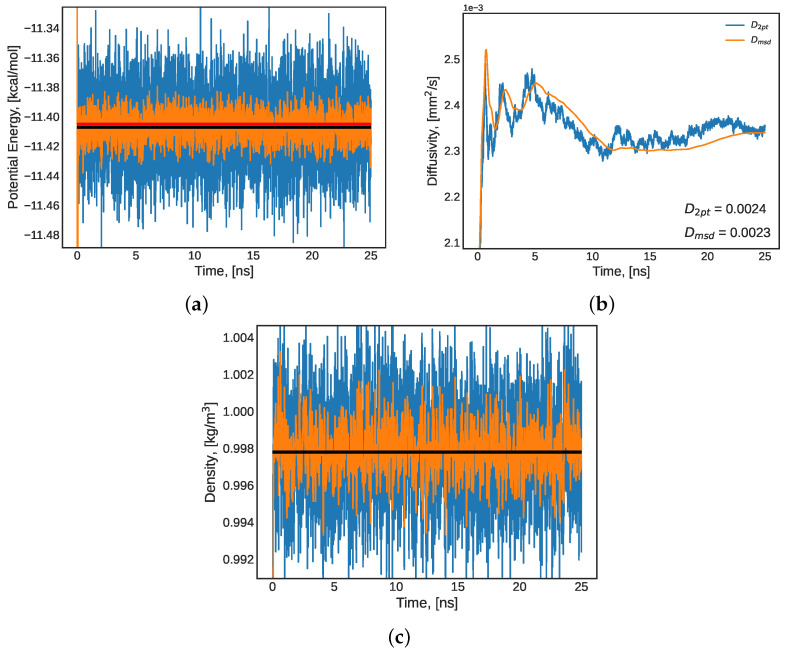
Pure water system at 25 °C and atmospheric pressure (**a**) potential energy (red: literature [80] value, black: mean value); (**b**) diffusivity; and (**c**) density (black: mean value) during 25 ns of an NPT ensemble equilibration simulation. In panels (**a**,**c**): blue: raw LAMMPS output, orange: average over a 0.4 ns running window.

This work used a TIP4P/2005 pure water system at 25 °C and atmospheric pressure as a model to demonstrate the equilibration procedure performed for all simulations presented here. As described above, the system was first simulated for 25 nanoseconds in an NPT ensemble, where the potential energy, diffusivity, and density of the system were observed to ensure that they approached the values presented by the original TIP4P/2005 water force field literature [80,81]. The simulation time-series results are presented in Figure 1 and the property values are compared to literature values in summary Table 3. The results presented indicated that during the NPT simulation, the system was approaching the literature values for density, potential energy, and diffusivity. These are properties utilized in the original parametrization of the TIP4P/2005 water force field [80], and thus serve as significant validation indicating an approach to equilibrium and physically meaningful results.

**Figure 2:**
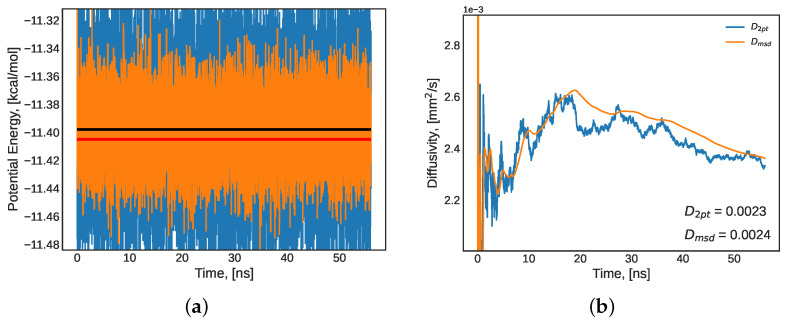
A pure water system at 25 °C and atmospheric pressure: (**a**) potential energy (red: literature [80] value, black: mean value) and (**b**) diffusivity during the last 50 ns segment of a 100 ns NVT equilibration. In panels (**a**,**c**): blue: raw LAMMPS output, orange: average over a 0.4 ns running window.

Once the equilibrium indications from Figure 1 and Table 3 were established, the system was released from its barostat and simulated in a canonical (NVT) ensemble for 100 ns. During this stage in equilibration, the mechanical disturbances from the volume corrections of the barostat were removed and the same properties (potential energy and diffusivity) were monitored and compared to literature values [80] to determine how approach to equilibrium. The results are presented in Table 3 and Figure 2. The time-series of potential energy and density presented in this section have the raw simulated values presented in blue (high noise signal), a running average presented in orange (attenuated noise), the mean value presented as a black horizontal line, and the literature value presented as a red horizontal line (Figure 1a,c and Figure 2a). The diffusivity plots (Figure 1 and Figure 2b) present the Einstein formulation of diffusivity (Equation (5)) through two methods of computing the mean squared displacement (MSD) slope—initial and final two-point slope (blue), and the running mean of all MSD values (orange). Both methods result in similar prediction performance relating to the literature value of water diffusivity under these conditions.

#### 3.1.2. Long-Term Equilibrium Indicators

**Figure 3:**
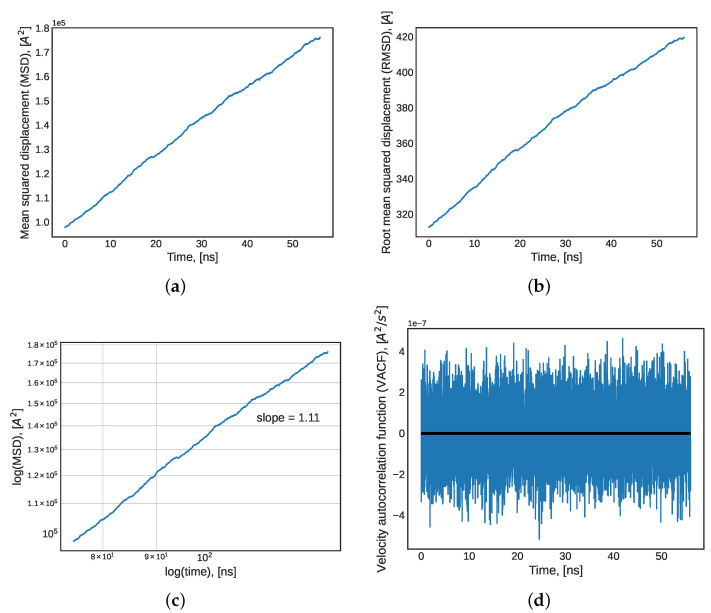
A pure water system at 25 °C and atmospheric pressure: (**a**) mean squared displacement (MSD); (**b**) root mean squared displacement (RMSD); (**c**) log-log plot of MSD; and (**d**) velocity time autocorrelation function (VACF) during the last 50 ns segment of a 100 ns NVT equilibration.

The physical properties predicted by MD used as indicators discussed above were further corroborated by long-term equilibrium indicators. All indicators presented in this section refer to whether the simulation was run for a sufficiently long time for all molecules to traverse (sample) the entire phase-space of the system. In other words, long-term indicators were used to determine whether the system reached the diffuse regime—at which point the system can be confidently used for transport property predictions. Furthermore, a proper simulation length can overcome periodic boundary condition discrepancies in molecular simulations. Firstly, the MSD of the simulation was compared to the box length (L) of the simulated region. In the diffuse regime, the MSD is much larger than L. Alternatively, the root mean squared displacement (RMSD) can be used: if it is larger than half of the box length (L/2), then the system is diffuse. Additionally, the slope of the log-log plot of MSD should be close to unity in the molecular diffuse regime [42]. Figure 3a presents the MSD, Figure 3b shows the RMSD, and Figure 3c presents the log-log plot for the NVT equilibration run for the model TIP4P/2005 water system. The system’s RMSD calculated at the end point of the NVT simulation presented in Figure 3b (approximately 420 angstroms) is one order of magnitude larger than half the longest dimension of the simulation box (84 Å), and the slope of the log-log plot is 1.11. Both of these results indicate that a diffuse regime was achieved. Then, the velocity autocorrelation function (VACF) was analyzed. The VACF of a well-sampled simulation in which the lag time between correlated velocities is not too large will converge to zero [42]. Additionally, the VACF can be used in the Green–Kubo formulations for transport property calculations, so it is important to ensure that the VACF converges towards zero so as to not introduce errors into the Green–Kubo integral. Figure 3d presents the NVT simulation’s VACF and its mean value converging to zero. This served as an added indicator of equilibration.

**Figure 4:**
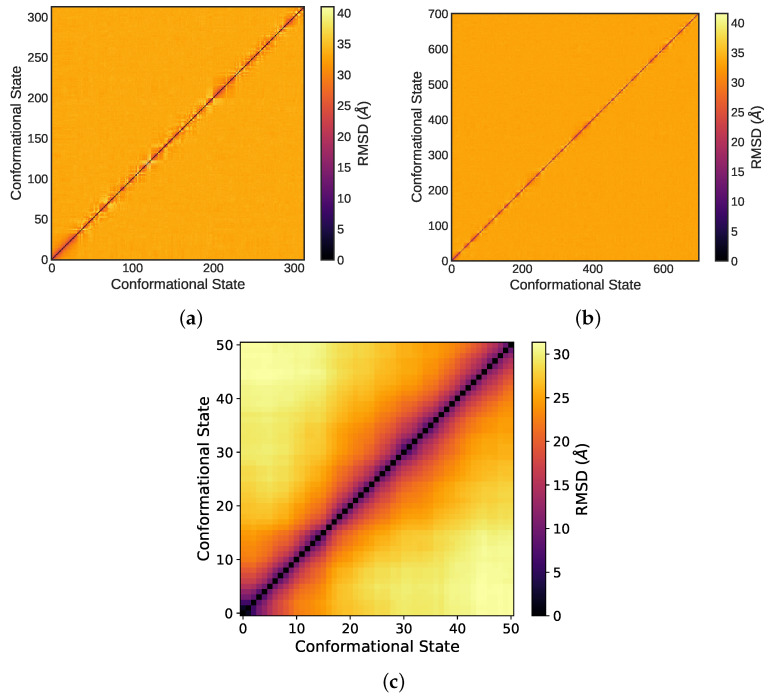
A pure water system at 25 ℃ and atmospheric pressure: (**a**) 25 ns NPT ensemble runs and (**b**) last 50 ns segment of a 100 ns NVT equilibration following the NPT run. Panel (**c**) presents the all-to-all RMSD plot of a simulation that is further from equilibrium.

A limitation of the MSD and RMSD indicators discussed above is that they are one-dimensional in nature. Although they quantify the diffusion of molecules through the simulation box, they do so by taking 3-dimensional motion and projecting the motion onto a one dimensional space. A more robust measure of the equilibration and presence of a diffuse regime is the all-to-all RMSD plot [93], which takes into consideration all three dimensions in molecular trajectories. In this plot, all conformational states from the molecular trajectory produced by the MD simulation are compared to all other conformational states by quantifying the RMSD between the states in units of distance. This plot measures how far apart each conformational state is from all others. By definition, the diagonal is always equal to zero, as it is equivalent to a conformational state compared to itself—and its distance from itself is zero. As the distance value increases, this signifies that the two conformational states compared are further apart from each other. Diffuse regime simulations have homogeneous all-to-all RMSD plots with an increased RMSD between states as moving away from the diagonal of the plot. Figure 4 presents the RMSD all-to-all plots for the NPT and NVT in the model TIP4P/2005 system. Figure 4c represents an unequilibrated system in which regions of conformational change are detected by the darker square regions that form on the plot. These correlate to smaller RMSD distances (see colorbar legend), and thus conformational states that have not yet traversed far from one another (not yet diffuse enough). In the case of the NVT ensemble (Figure 4b), the diagonal is thin and no regions of low RMSD values (darker colors) are evident. This indicates a diffuse regime and approach to equilibrium.

Finally, the Stokes–Einstein constant was calculated and temperature-normalized at 25 °C (298 K) for the quantification of each simulated system’s deviation from ideal behaviour. The largest deviation was determined to be 9.1% at 0 °C. Additionally, pressure did not have an effect on the Stokes–Einstein fidelity in the methane gas hydrates systems simulated. Deviations arising from the use of the TIP4P/2005 water model have been previously reported, but these violations were more pronounced at colder temperatures (below 260 K) as the system approaches water’s liquid–liquid critical point near its Widom line [40]. The deviations observed from the simulations performed in this work did not indicate abnormal behaviour that would lead to concern about the equilibration process described above.

#### 3.1.3. Hydrogen Bonding Effects

Hydrogen bond effects are known to be a dominant factor for molecular transport in aqueous systems [94], and a major contributor to the intermolecular forces that give rise to the bulk property of dynamic viscosity [95,96,97]. Recently, H-bond vibrations were indirectly measured by determining the stretching vibration of OH covalent bonds in water via infrared absorption in an effort to experimentally determine viscosity [98]. The importance of hydrogen bonding as a contributor to viscosity must be considered in the design of molecular simulations for transport property predictions. For this reason, this work implements an all-atom (AA) version of the OPLS water force fields considered here (TIP4P/2005 and TIP4P/Ice). This normally renders the simulation more computationally intensive. However, if the intention is the prediction of transport properties in systems known to be dominated by hydrogen bonds (water and gas hydrates), then coarser-grained force field variations such as the OPLS united atom (UA) would be inappropriate as they fail to fully model H-bond interactions. Furthermore, it is this increased complexity in all-atom MD systems that enhances the necessity for a robust equilibration procedure, as presented in this section, to ensure that such simulated systems are properly designed and physically representative.

### 3.2. Water Models

#### 3.2.1. Viscosity

**Figure 5:**
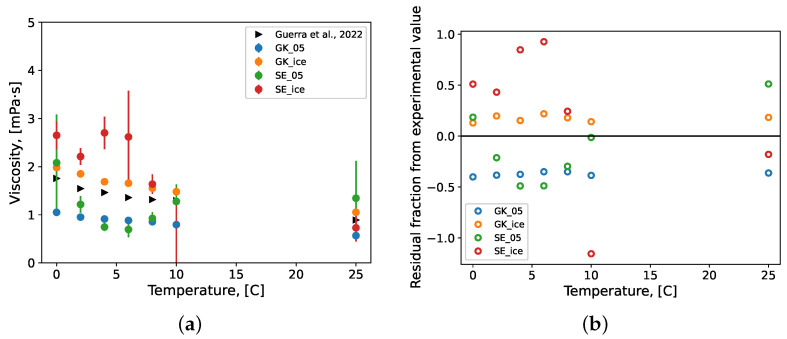
The (**a**) viscosity of pure water systems and (**b**) fractional residual difference between the MD prediction and experimental value. Repeated simulation produced samples of each condition’s prediction value of size *n* = 10; theses samples were bootstrapped with replacement (*n* = 10,000) to calculate the mean values which are presented here with their standard error bars. Experimental values were collected and presented elsewhere by Guerra et al. [30]. GK: Green–Kubo formulation; SE: Stokes–Einstein formulation, 05: TIP4P/200; ice: TIP4P/Ice.

The bootstrapped values of the viscosity predictions from the molecular simulations performed here are presented in Figure 5a with their standard errors from the bootstrap analysis. The experimental values of dynamic viscosity for pure water systems, which were measured and presented elsewhere [30], were also added (black triangles) for comparison. Figure 5b shows the deviation between prediction values and the experimental data in the form of residual difference fractions. The dynamic viscosity was predicted using the Green–Kubo (Equation (2)) and the Stokes–Einstein (Equation (7)) formulations presented above. The TIP4P/Ice predictions overestimated the experimental values and the TIP4P/2005 predictions, except for at 25 °C, where the TIP4P/Ice model underestimated both. This behaviour is expected as at low temperatures, this force field more closely models solid water. The Green–Kubo (GK) prediction resulted in more stable viscosity predictions across temperatures than the Stokes–Einstein (SE) predictions. This is evident from the lower GK standard errors (Figure 5a) and the distribution of residual differences in Figure 5b. The SE predictions deviated more from experimental values near 5 °C, which is near the maximum density point for water (at 4 °C). Additionally, the SE prediction results in larger standard errors as the SE formulation (Equation (7)) is inversely proportional to MSD and thus sensitive to changes in density. Increased density leads to greater impedance in molecular displacement, and thus higher viscosity (Equation (7)). The variance in standard error between consecutive temperature conditions can be attributed to the bootstrapping procedure described above, where different random-number generator seeds are used to populate molecular velocities and thus set temperature. For these reasons, the SE predictions were determined to not offer an improvement on the GK predictions in the water systems simulated here. Finally, due to the improved performance of the TIP4P/Ice force field in the context of dynamic viscosity predictions at lower temperatures, it was selected as the most appropriate between the two to be used to model gas hydrate systems (similar to ice forming conditions) in the remainder of this work.

#### 3.2.2. Diffusivity and Thermal Conductivity

**Figure 6:**
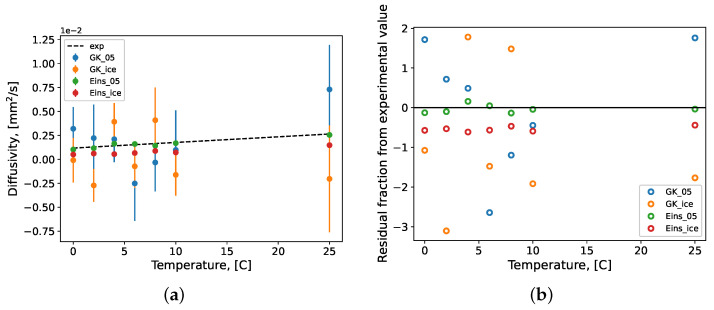
The (**a**) diffusivity of pure water systems and (**b**) fractional residual difference between MD prediction and experimental value. Repeated simulation produced samples of each condition’s prediction value of size *n* = 10; theses samples were bootstrapped with replacement (*n* = 10,000) to calculate mean values which are presented here with their standard error bars. GK: Green–Kubo formulation; Eins: Einstein formulation; 05: TIP4P/200; ice: TIP4P/Ice; exp: linear regression of experimental data [99,100,101,102,103].

The diffusivity of the water models simulated here are presented in Figure 6. Literature experimental data were used to develop a linear regression for the temperature range considered [99,100,101,102,103]. It is evident from Figure 6 that the Einstein formulation for diffusivity was the best performing prediction for this property regardless of the water force field used. This formulation benefits from the stability of the Einstein slope over the Green–Kubo autocorrelation integral [42], which is more unstable as seen by the increased deviations between the GK predictions and the experimental regression in Figure 6b. The Einstein prediction for the TIP4P/2005 system closely follows (−13–15% difference) the experimental linear regression culminating at a relatively accurate value of 25 °C, underestimating the accepted value by only 3.9%. This is expected as diffusivity was one of the properties used in the development of the TIP4P/2005 force field parameters [80]. Figure 6b depicts a deviation from the overall trend in Einstein diffusivity predictions for both water models near the density maximum of water (4 °C). Due to the use of the Einstein slope in the Stokes–Einstein formulation of viscosity, these deviations in diffusivity lead to deviations in the viscosity predictions discussed above at the same temperature.

**Figure 7:**
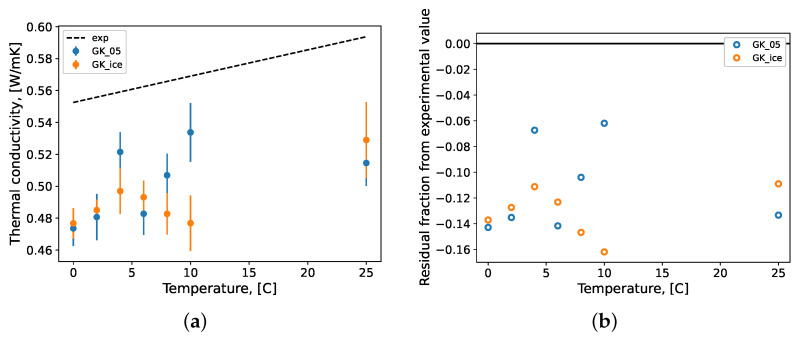
The (**a**) thermal conductivity of pure water systems and (**b**) fractional residual difference between MD prediction and experimental values. Repeated simulation produced samples of each condition’s prediction value of size *n* = 10; these samples were bootstrapped with replacement (*n* = 10,000) to calculate the mean values which are presented here with their standard error bars. GK: Green–Kubo formulation; 05: TIP4P/2005; ice: TIP4P/Ice; exp: linear regression of experimental data [104,105].

The thermal conductivity predictions for the water systems are presented in Figure 7. The experimental data presented are a linear regression from thermal conductivity data gathered elsewhere [104,105]. The thermal conductivity was calculated using the GK formulation (Equation (4)) for both water force fields. Although somewhat unstable, the GK formulation for thermal conductivity produced more accurate values than the GK formulation for viscosity, relative to their respective experimental data. This is likely due to the higher fluctuation in the pressure tensor elements (Equation (2)) versus heat flux (Equation (4)) in an NVT ensemble simulation, where temperature is controlled dynamically. Both the TIP4P/2005 and TIP4P/Ice models performed similarly in the prediction of thermal conductivity, with a percentage difference from experimental data regression, minimally of −6% and maximally of −16%.

### 3.3. Methane Gas Hydrate Systems

**Figure 8:**
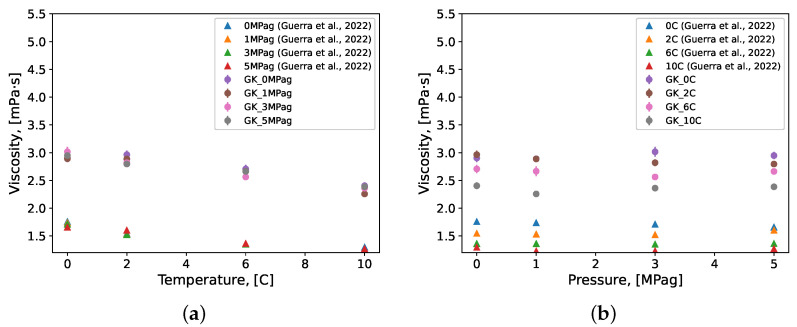
Viscosity of the methane gas hydrate systems as simulated by MD under pre-nucleation conditions using the TIP4P/Ice water force field model. Viscosity is presented as a function of (**a**) temperature and (**b**) pressure. Repeated simulation produced samples of each condition’s prediction value of size *n* = 10; these samples were bootstrapped with a replacement (*n* = 10,000) to calculate mean values which are presented here with their standard error. Experimental values were collected and presented elsewhere by Guerra et al. [30]. GK: Green–Kubo formulation.

The viscosity of the methane gas hydrate systems under pre-nucleation conditions were predicted using the same procedure as for the pure water systems discussed above. An overestimation in dynamic viscosity predicted by the molecular simulations for all conditions considered was evident from the results (Figure 8). The TIP4P/Ice force field was parametrized with the intent of representing the solid phase properties of ice [81]. It is then unsurprising that the MD simulations implementing this force field overestimated the dynamic viscosity of sub-cooled water as the force field generally has a higher fidelity to the properties of the solid phase water. This overestimation was evident in the pure water systems presented above (Figure 8a). However, the results presented here show this overestimation to be exacerbated in the presence of methane molecules. MD simulations overestimated the experimental values of viscosity at a minimum by 65% (0 °C and 0 MPag), and by a maximum of 99% (6 °C and 1 MPag). The overall mean overestimation of dynamic viscosity by the MD simulations was approximately 84% of the experimental values presented.

In light of the robust equilibration procedure, simulation design considerations, and statistical analysis of the results presented above, this work indicates an inherent limitation to the development of physically representative MD simulations in the context of transport property estimations of methane gas hydrate systems. This limitation may be addressed with the development a new water force field with the intent of predicting the transport properties of pre-nucleation and nucleating condition methane gas hydrates systems. The experimental data of such systems should be used in the parametrization of the new force field. Due to the improved performance of the TIP4P/Ice over the TIP4P/2005 force field in the pure water systems considered above, the authors suggest a re-parametrization of the TIP4P/Ice as a starting point. However, although the differences between MD predictions using the current force field and experimental measurements may be large in relative terms, the absolute differences may be negligible for application purposes depending on process specifics, such as scale and/or geometry. The results presented by this work indicate that scientists and engineers may be informed by MD simulations about methane hydrate systems’ bulk viscosity using the existing TIP4P/Ice force field, if care is taken to avoid the general and absolute conclusions from the results. Simulations may provide useful information when compared to one another, and this work can serve as a useful guide for translating the deviation of MD predictions of viscosity to experimental measurements.

**Figure 9:**
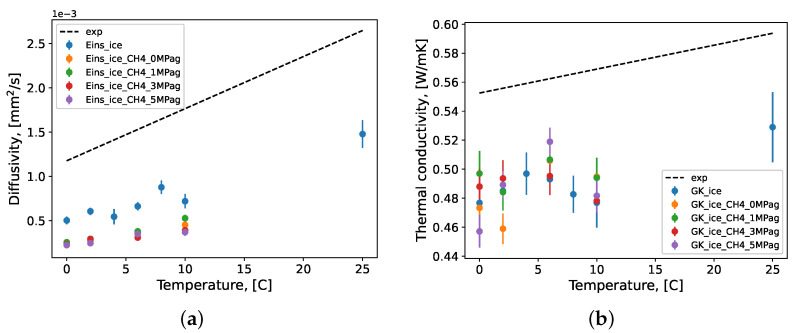
The (**a**) diffusivity and (**b**) thermal conductivity of the methane gas hydrate systems as simulated by MD under pre-nucleation conditions using the TIP4P/Ice water force field model. Repeated simulations produced samples of each condition’s prediction value of size *n* = 10; these samples were bootstrapped with replacement (*n* = 10,000) to calculate mean values, which are presented here with their standard error. GK: Green–Kubo formulation; Eins: Einstein formulation; ice: TIP4P/Ice; exp: linear regression of experimental data for panel (**a**) [99,100,101,102,103]; exp: linear regression of experimental data for panel (**b**) [104,105].

The diffusivity and thermal conductivity of methane gas hydrate systems were calculated in the molecular simulations performed in this work. There were not, however, direct experimental data obtained at the desired conditions for comparison. In light of this, these transport properties are presented for completeness and no general conclusions were drawn. However, the inadequacy of the simulations to predict viscosity would indicate inadequacy in its capability to predict diffusivity, as these properties are physically closely related. The results from the bootstrap procedure from the repeated production NVT ensemble simulations are presented in Figure 9. Both the diffusivity and the thermal conductivity were simulated at various pressures, which are presented in this figure. The same linear regressions of the experimental data for pure water from Figure 6 and Figure 7a were also included in Figure 9 as a reference point.

### 3.4. Hydrogen Bond Analyses

The important role played by H-bonds in the context of the transport properties of aqueous systems has been discussed above. In light of this, the simulations performed here were designed with the OPLS-AA force fields in which hydrogen atom interactions were explicitly and independently treated, and were not lumped into united atom (UA), or other coarse grain, representations. This type of hydrogen model enabled an extensive H-bond analysis to be performed on the molecular dynamics systems to further elucidate the overestimated viscosity results presented above. For all simulated systems, the H-bond lifetime, the average number of H-bonds per donor molecule available, and the average number of H-bonds over time were determined. Additionally, H-bond length and angle probability density distributions were produced. All H-bonds in this work were identified using the Python package MDAnalysis. The geometric criteria that defined an H-bond were (1) donor–acceptor distance of less than 3 Å; and (2) donor–hydrogen–acceptor angle greater than 150 degrees. The results for pure water systems were summarized in Table 4 and Table 5.

The hydrogen bond lengths and angles were determined via a Gaussian kernel density estimation, which provided the probability density functions (PDFs) for both variables. The most probable (highest density) values of the PDFs were presented in Table 4. The summary tables demonstrate that, in the considered range, temperature did not have a significant effect on the most probable H-bond lengths and angles. However, an increase in the average number of H-bonds over time did result from decreased temperatures for both force fields. Moreover, the percentage differences between the values obtained from the TIP4P/Ice compared to the TIP4P/2005 were almost negligible—at a maximum of 0.72% for bond length, and 0.91% for bond angle. Conversely, the difference between force fields for the average number of H-bonds per donor molecule and the average H-bonds over time were determined to be non-negligible −5.81–7.78% and 5.84–7.54%, respectively, (Table 5). Additionally, it is evident that the differences between force fields for the average number of H-bonds over time and per donor molecule reached maximums near the density maximum point for water (4 °C).

Similar results were obtained for the methane systems simulated—temperature did not have an effect on the most probable H-bond length and angle, nor on the average number of H-bonds per donor molecule (Table 6). In addition to the water molecules, these systems had one more than one donor molecule type available to form H-bonds—methane. The added donor molecule resulted in a 6.7–7.8% higher number of average H-bonds over the simulated time compared to the pure water system modeled by the same water force field (TIP4P/Ice). Additionally, in the range considered by this work, pressure did not have a considerable effect on any of the H-bond quantities discussed above for the methane systems studied (Table 6).

This work also determined the hydrogen bond lifetimes for the systems simulated. This analysis was based on the autocorrelation of the binary state (1 or 0) of an H-bond (present or not present) between all possible donor and acceptor atoms at each time step of the molecular simulation. The H-bond lifetime autocorrelation has been described elsewhere [106,107]. This was performed using MDAnalysis Python package with trajectories from equilibrated production runs output every 2 femtoseconds for 20 picoseconds. The autocorrelation data were fit to an exponential model to determine the time constant of the H-bond lifetime. The time constant was presented in Table 5 and Table 6. In pure water systems, the H-bond lifetimes for the TIP4P/Ice water force field were between 13.8 and 14.6% longer than those for the TIP4P/2005 force field (Table 5). Moreover, the lifetimes for the methane hydrate TIP4P/Ice systems were between 16.8 and 19.2% higher than for the pure water system with the same force field. In both cases, the increased H-bond time constant coincided with a higher number of hydrogen bonds over the simulated time, and with increased estimations of viscosity.

The discrepancy in the viscosity predictions between the TIP4P/2005 versus the TIP4P/Ice force fields discussed above (Figure 5) can be attributed to the different behaviour of H-bonds between force fields. The original re-parametrization of TIP4P/2005 to develop the TIP4P/Ice force field [81] may have strengthened the H-bond interactions, leading to longer H-bond lifetimes and thus a larger average number of H-bonds over time. This had the effect of increasing the estimated viscosity of pure water at lower temperature, and thus improved the viscosity predictions over those obtained by the original TIP4P/2005 force field at these temperatures (Figure 5). However, in the presence of an added hydrogen donor that can contribute hydrogen bonds in same order of magnitude as water (evident from the per molecule number of H-bonds in Table 6), the TIP4P/Ice hydrogen interactions may have become too dominant and led to an overestimation of the bulk viscosity of the methane hydrate systems simulated in this work. The nature of any future re-parametrization of the TIP4P/Ice force field for the purpose of methane hydrate applications is out of the scope of the current work and it is not discussed here. Thus, the evidence to the role of hydrogen bonds to the viscosity overestimation was presented in this section to inform future attempts to correct this behaviour in all-atom methane hydrate molecular dynamics systems.

## 4. Conclusions and Future Work

This work examined two OPLS all-atom force field water models (TIP4P/2005 and TIP4P/Ice) in an attempt to assess the predictions of transport properties from MD simulations of methane gas hydrates systems under pre-nucleation conditions. Their predictions were directly compared against experimental data. The TIP4P/2005 force field was determined to have improved performance over TIP4P/Ice in predicting the diffusivity of pure water systems under the conditions examined. However, the predictions of viscosity by the TIP4P/Ice force field had improved performance as evidenced by lower residuals from experimental values. Based on this result, the TIP4P/Ice force field was determined to be more adequate to model sub-cooled water conditions for the simulation of methane gas hydrate systems in the context of predicting dynamic viscosity. The viscosity of various methane gas hydrate systems under pre-nucleation conditions was estimated by MD and directly compared to previous experimental results. The Stokes–Einstein formulation for viscosity was shown to not provide improvements in prediction at lower temperatures as it suffers from the instability of the Einstein slope at the water density maximum (4 °C).

Regarding the methane hydrate systems, the MD viscosity predictions followed experimental temperature and pressure trends, but overestimated experimental measurements by 84% on average. Hydrogen bond analyses were performed to further characterize the molecular sources of this large deviation. Differences in H-bond length and angle across temperatures and between force fields were determined to be negligible (0.72–0.92%). However, the differences between force fields for the average number of H-bonds over time and per donor molecule were shown to be non-negligible (5.81–7.78%). Additionally, the H-bond lifetimes for the TIP4P/Ice force field were determined to be 13.8–14.6% longer than those resulting from the TIP4P/2005 systems. When methane was added to the system, H-bond lifetimes increased between 16.8 and 19.2% over pure water systems with the same force field. The H-bond analyses presented in this work indicate possible sources for the viscosity overestimation in the case of the methane systems presented here.

Although the differences between MD predictions and experimental measurements may be large in relative terms, the absolute difference between predictions may be negligible for application purposes. The results presented by this work indicate that viscosity information may be used from MD simulations using the TIP4P/Ice force field, if care is taken to avoid drawing general and absolute conclusions from the results. Simulations may provide useful information when compared to one another, and this work can serve as a useful guide for relating MD predictions to experimental measurements.

Overall, the results presented herein indicate an inherent limitation to MD simulations predicting the transport properties of methane gas hydrate systems. Future work should attempt to address these limitations to improve the accuracy of molecular simulations in this context. A re-parametrization of the TIP4P/Ice water model for atmospheric and increased pressures for methane gas hydrate applications may be the next logical step. This work may serve as a baseline or guidance for future re-parametrization efforts, or for the development of alternative solutions. If MD is to be an accurate tool for the dynamic viscosity prediction of methane gas hydrate systems under pre-nucleation conditions, the experimental data utilized here and presented elsewhere [30] should be used in any efforts to address this limitation.

## Figures and Tables

**Table 1:** LAMMPS force field parameters for various OPLS-AA water models.

Attribute, Units	TIP4P [77]	TIP4P/2005 [80]	TIP4P/Ice [81]
O mass, g/mol	15.9994	15.9994	15.9994
H mass, g/mol	1.008	1.008	1.008
O charge, e	−1.04	−1.1128	−1.1794
H charge, e	0.52	0.5564	0.5897
OH bond $r_{o}$, Å	0.9572	0.9572	0.9572
HOH angle θ	104.52°	104.52°	104.52°
OM distance, Å	0.15	0.1546	0.1577
O-O LJ ϵ, kcal/mol	0.155	0.1852	0.21084
O-O LJ σ, Å	3.1536	3.1589	3.1668
O-H LJ ϵ, kcal/mol	0	0	0
O-H LJ σ, Å	0	0	0
H-H LJ ϵ, kcal/mol	0	0	0
H-H LJ σ, Å	0	0	0
$r_{c}$, Å	8.5	8.5	8.5

Notes: H: hydrogen; O: oxygen; O-O: oxygen–oxygen interactions; H-H: hydrogen–hydrogen interactions; charge units in multiples of an electron charge (e); M is the massless fourth site in the TIP4P water model; *r_c_*: cutoff distance of Coulombic and Lennard-Jones (LJ) interactions, *σ*: distance for zero potential energy in LJ potential, *ϵ* is the depth of the LJ potential well.

**Table 2:** LAMMPS force field parameters for OPLS-AA methane model.

Attribute, Units	OPLS-AA [79]
C mass, g/mol	12.011
H mass, g/mol	1.008
C charge, e	−0.24
H charge, e	0.06
CH bond $r_{o}$, Å	1.09
CH angle θ	107.8°
C-C LJ ϵ, kcal/mol	0.066
C-C LJ σ, Å	3.5
H-H LJ ϵ, kcal/mol	0.03
H-H LJ σ, Å	2.5
C-H LJ ϵ, kcal/mol	0
C-H LJ σ, Å	0
$r_{c}$, Å	8.5

Notes: H: hydrogen; O: oxygen; O-O: oxygen–oxygen interactions; H-H: hydrogen–hydrogen interactions; charge units in multiples of an electron charge (e); M is the massless fourth site in the TIP4P water model; *r_c_*: cutoff distance of Coulombic and Lennard-Jones (LJ) interactions, *σ*: distance for zero potential energy in LJ potential, *ϵ* is the depth of the LJ potential well.

**Table 3:** A summary of the system’s mean physical properties after the NPT and NVT equilibration simulations and a comparison to expected literature values for a TIP4P/2005 pure water system at 25 °C and atmospheric pressure.

Property, Units	TIP4P/2005 Literature [80]	NPT (25 ns)	NVT (100 ns)
Density, kg/m^3^	0.998	0.997	0.992
Potential Energy, kcal/mol	−11.405	−11.407	−11.398
Diffusivity, mm^2^/s	2.28 × 10${}^{-3}$	2.23 × 10${}^{-3}$	2.24 × 10${}^{-3}$

**Table 4:** Hydrogen bond lengths and angles at various temperatures and 0 MPag for pure water systems. Bond lengths and angles are based on a Gaussian kernel density estimation, and the values presented correspond to the highest density in each system. 2005: TIP4P/2005 [80]; Ice: TIP4P/Ice [81]; Diff = (Ice − 2005)/2005 × 100%.

T	Hydrogen Bond			Hydrogen Bond		
°C	Length, Å			Angle, Deg		
	2005	Ice	Diff, %	2005	Ice	Diff, %
	±0.005	±0.005	±0.005	±0.005	±0.005	±0.005
0	2.77	2.77	0.00	165.35	166.86	0.91
2	2.76	2.78	0.72	166.75	166.65	−0.06
4	2.77	2.78	0.36	166.45	167.16	0.43
6	2.77	2.79	0.72	166.45	166.95	0.30
8	2.76	2.78	0.72	165.65	166.45	−0.12
10	2.76	2.78	0.72	165.85	167.36	0.91
25	2.76	2.78	0.72	165.95	166.25	0.18

**Table 5:** Average number of hydrogen bonds (HBs) per donor molecule, the average number of HBs over the simulated time, and the time constants (τ) from an exponential fit of HB lifetimes at various temperatures and 0 MPag for pure water systems. 2005: TIP4P/2005 [80]; Ice: TIP4P/Ice [81]; Diff = (Ice − 2005)/2005 × 100%.

T	HB			Avg. Number of			HB Lifetime Time		
°C	per Molecule			HB over Time			Constant (τ), ps		
	2005	Ice	Diff, %	2005	Ice	Diff, %	2005	Ice	Diff, %
	±0.005	±0.005	±0.005	±0.5	±0.5	±0.005	±0.005	±0.005	±0.005
0	2.59	2.75	6.18	3292	3507	6.53	5.27	6.03	14.52
2	2.57	2.76	7.39	3131	3356	7.19	5.26	6.02	14.50
4	2.57	2.77	7.78	2825	3038	7.54	5.20	6.03	16.09
6	2.57	2.75	7.00	2483	2656	6.97	5.26	6.04	14.80
8	2.58	2.73	5.81	2174	2301	5.84	5.27	6.00	13.85
10	2.58	2.75	6.59	1899	2022	6.47	5.25	5.59	14.16
25	2.57	2.74	6.61	1887	2017	6.89	5.23	6.00	14.60

**Table 6:** Bond lengths and angles from a Gaussian kernel density estimation, the average number of hydrogen bonds (HBs) per hydrogen donor available (H_2_O and CH_4_), and time constants (τ) from an exponential fit of HB lifetimes for methane hydrate systems simulated using the TIP4P/Ice force field.

T	P	Length	Angle	HB	HB	HB	τ
°C	MPag	Å	Degree	N/H_2_O	N/CH_4_	N	ps
		±0.005	±0.005	±0.005	±0.005	±0.5	±0.005
0	0	2.77	166.85	2.97	2.65	3779	7.18
2	0	2.77	167.66	2.96	2.95	3603	7.17
6	0	2.77	167.85	2.95	3.16	2847	7.09
10	0	2.77	166.95	2.93	2.85	2157	7.00
0	1	2.77	167.56	2.99	2.95	3806	7.30
2	1	2.77	167.46	2.96	2.92	3601	7.17
6	1	2.77	166.85	2.95	3.16	2850	711
10	1	2.76	166.95	2.94	2.57	2164	6.99
0	3	2.77	167.25	2.97	2.89	3788	7.18
2	3	2.77	167.36	2.98	2.95	3596	7.16
6	3	2.76	167.76	2.96	3.04	2862	7.18
10	3	2.76	167.06	2.94	2.68	2161	6.99
0	5	2.77	167.96	2.98	3.05	3800	7.23
2	5	2.77	168.16	2.95	2.90	3590	7.09
6	5	2.77	167.56	2.95	2.85	2853	7.09
10	5	2.77	166.95	2.95	3.16	2174	7.10

## Data Availability

Not applicable.

## Sample Availability

Samples of the compounds are available from the authors.

## Abbreviations

The following abbreviations are used in this manuscript:

AA	All-atom
DFT	Density functional theory
Eins	Einstein
EOS	Equation of state
GK	Green–Kubo
H-bond	Hydrogen bond
LAMMPS	Large-scale Atomic/Molecular Massively Parallel Simulator
LJ	Lennard-Jones
MD	Molecular dynamics
MSD	Mean squared displacement
NPT	Isothermal-isobaric ensemble
NVT	Canonical ensemble
OPLS	Optimized potentials for liquid simulations
OPLS-AA	Optimized potentials for liquid simulations all-atom
OPLS-UA	Optimized potentials for liquid simulations united-atom
PDF	Probability distribution function
RMSD	Root mean squared displacement
SE	Stokes–Einstein
TIP4P	Transferable intermolecular potential with 4 points
UA	United atom
VACF	Velocity autocorrelation function
